# A mid-morning snack of almonds generates satiety and appropriate adjustment of subsequent food intake in healthy women

**DOI:** 10.1007/s00394-014-0759-z

**Published:** 2014-09-03

**Authors:** Sarah Hull, Roberta Re, Lucy Chambers, Ana Echaniz, Martin S. J. Wickham

**Affiliations:** 1 Nutrition Research, Leatherhead Food Research, Randalls Road, Leatherhead, Surrey, KT22 7RY UK; 2 School of Psychology, Pevensey Building, University of Sussex, Brighton, BN1 9QH UK

**Keywords:** Almonds, Satiety, Appetite, Snack, Energy intake, *Ad libitum*, Visual analogue scales (VAS)

## Abstract

**Purpose:**

To assess the effect of consuming a mid-morning almond snack (28 and 42 g) tested against a negative control of no almonds on acute satiety responses.

**Method:**

On three test days, 32 healthy females consumed a standard breakfast followed by 0, 28 or 42 g of almonds as a mid-morning snack and then ad libitum meals at lunch and dinner. The effect of the almond snacks on satiety was assessed by measuring energy intake (kcal) at the two ad libitum meals and subjective appetite ratings (visual analogue scales) throughout the test days.

**Results:**

Intake at lunch and dinner significantly decreased in a dose-dependent manner in response to the almond snacks. Overall, a similar amount of energy was consumed on all three test days indicating that participants compensated for the 173 and 259 kcals consumed as almonds on the 28 and 42 g test days, respectively. Subjective appetite ratings in the interval between the mid-morning snack and lunch were consistent with dose-dependent enhanced satiety following the almond snacks. However, in the interval between lunch and dinner, appetite ratings were not dependent on the mid-morning snack.

**Conclusion:**

Almonds might be a healthy snack option since their acute satiating effects are likely to result in no net increase in energy consumed over a day.

## Introduction

Satiety—the inter-meal inhibition of hunger and eating that arises as a result of consuming food [1]—is influenced by a wide variety of interacting factors, involving physiological processes in the brain and body, and the social and physical environments [2]. Foods that generate strong sensations of satiety can help consumers control their appetite, eat healthily and manage their weight [3]. A problem for weight management is thought to be snacking [4]. This eating habit is commonplace [5] and likely to add calories to a person’s total daily energy intake if the consumed snack food has little impact on satiety, resulting in poor adjusted intake at their next meal(s). Therefore, it is important to identify healthy satiating snack foods that support appropriate calorie-dependent adjustment of subsequent intake, so that snacking is less likely to result in a net increase in energy consumed.

It is well established that calorie-for-calorie not all foods deliver the same level of satiety [6]. For example, in satiety studies where comparison foods were matched for energy content, there is considerable evidence that high-protein foods are more satiating than those that are high in carbohydrate and/or fat [7]; that fibre-rich foods are more satiating than low-fibre foods [8]; and that energy-dense foods are less satiating than those with lower energy density [9].

Whole almonds have a nutritional profile consistent with satiety, being the tree nut highest in protein and fibre. Additionally, they have other health benefits because they are a good source of vitamin E, riboflavin, niacin, calcium, magnesium and potassium [10]. However, almonds are also a high-fat energy-dense food; these types of foods might be an inappropriate snack choice since when eaten in the same volume as low-energy-dense foods they are equally as satiating but higher in energy [9].

There is some evidence that consuming almonds can have positive effects on appetite control. Long-term studies indicate that almonds do not lead to significant changes in body weight [11–14]; this might be because habitual consumption of almonds increases resting energy expenditure and/or because almonds have a high satiety value and people are able to appropriately compensate for their consumption [12]. In the short term, adding almonds to a meal has been reported to decrease blood glucose concentrations and increase satiety in adults with impaired glucose tolerance [15], with similar glycaemic results reported for healthy individuals [16, 17]. In a recent study, 250 kcal of almonds as a snack reduced hunger and desire to eat at a subsequent meal in people with increased risk of type 2 diabetes, though intake at this meal was fixed and so compensation effects cannot be assessed [13]. One short-term study has examined the acute effects of almond intake on satiety in healthy people [18]; however, an unusually large portion of almonds was consumed (80 g: 500 kcals) and only self-reported measures were used to assess effects on satiety.

No acute studies have objectively assessed whether snacking on almonds leads to portion-size-dependent changes in subsequent food intake, and this is the aim of the present study. This information will shed light on whether the high satiety value of almonds is the reason why habitual snacking on this food results in insignificant changes in body weight over the longer term. This study measured the effects of an almond snack on satiety (appetite sensations and ad libitum intake) over a day. Two different test quantities of almonds were assessed: 28 and 42 g. These portions were selected to be typical of normal consumption in a free-living situation and to assess whether compensation behaviours were portion-size dependent. A no almond test day was included in the study as a negative control.

## Methods

### Study design

Two different portions of an almond mid-morning snack (28 and 42 g) were compared to a no almond control using a double Latin square randomised crossover design, with test day (0, 28 and 42 g almonds) as the within-subject measure. The main outcome measures of satiety were intake (kcal) at the two ad libitum test meals, and subjective appetite ratings of hunger and fullness [visual analogue scale (VAS) scores].

### Participants

Participants were recruited from Leatherhead Food Research’s volunteer database, and adverts were placed in papers, shops and companies in the local area. Participants had to meet the following inclusion criteria: female; age at start of the study ≥35 and ≤60 years; body mass index (BMI) ≥18.5 and ≤25 kg/m^2^; apparently healthy: no reported current or previous metabolic diseases or chronic gastrointestinal disorders; dietary habits: no medically prescribed diet, no slimming diet, used to eating three meals a day; no blood donation during the study; reported intense sporting activities ≤10 h/w; reported alcohol consumption ≤14 units/w; and informed consent signed. Participants also had not to meet any of the following exclusion criteria: smoking; vegetarian; dislike, allergic or intolerant to the test products; possible eating disorder [scoring >2 on SCOFF questionnaire [19] and/or scoring >14 on Revised Restraint Scale [20]]; reported medical treatment that may affect eating habits/satiety; and reported participation in another biomedical trial 1 month before the start of the study. Thirty-two participants were recruited and completed the study, although incomplete appetite ratings data for Interval 2 were obtained for one participant. Participant characteristics are described in Table 1.

**Table 1:** Participant characteristics

	Females
*N*	32
Age (years)	48.4 ± 1.0
BMI (kg/m^2^)	22.7 ± 0.26
Revised Restraint Scale Score	7.9 ± 0.59
SCOFF score	0.0 ± 0.03

The study was submitted to Surrey Research Ethics Committee and was granted a favourable ethical opinion (REC reference: 12/LO/0535). All participants gave written informed consent to participate in the study. The study was conducted in accordance with the ethical standards laid down by the 1964 Declaration of Helsinki and in accordance with Good Clinical Practice guidelines.

### Protocol

Over a period of 5 weeks, each volunteer visited the test facility on three occasions, with a 2-week washout period between each visit. The day before the study, participants were asked to consume their evening meal no later than 20.00 and asked to record everything they consumed on this day between 18.00 and 20.00. They were instructed to consume the same foods at the same time the evening prior to each subsequent test day. Participants were also asked to abstain from alcohol and vigorous exercise for 24 h prior to each test. Drinking after 20.00 was allowed but restricted to only water. Participants were asked to refrain from drinking any liquids for 1 h before the start of the study visit.

On each test day, participants were instructed to arrive at the Nutrition Unit at Leatherhead Food Research at 08.00. Participants remained in the Nutrition Unit for the duration of the study day. Between eating occasions, they were seated in volunteer rooms in a controlled environment and allowed to read or use laptop computers, but were not permitted to eat and drink between meals, with the exception of water. Water (up to 150 ml per hour) was allowed during the test day; however, participants were asked to abstain from drinking for 45 min before and after consumption of the test product. To ensure similar conditions existed during each test day, consumption of water on the first test day was recorded and repeated at each subsequent test day.

Immediately prior to consumption of breakfast, participants completed baseline appetite ratings. At 08.30 (*T* = −150), participants were provided with a breakfast that closely mimicked their usual consumption. They were given their habitual morning drink (tea/coffee/water) and instructed to drink all that was given (200 ml). Participants were seated in booths to isolate them from each other and given 15 min to consume the breakfast. Questions on satiety were then asked every 30 min until immediately prior to consumption of the mid-morning snack at 11.00 (*T* = 0) when participants received the test food (0, 28 or 42 g of almonds) to consume within 15 min. Water (100 ml) was provided with this snack, and participants who were given the control were also given the same quantity of water. Thereafter, questions on satiety were asked at 15-min intervals for 90 min, until immediately prior to consumption of the first ad libitum meal at 12.30 (*T* = 90). For the first course of this meal, participants received an ad libitum portion of ham and cheese sandwiches. Once they were comfortably full from the sandwiches, they were given an ad libitum portion of strawberry yogurt. Participants were given 30 min to consume the meal and were instructed to eat only until they were comfortably full. If they had not finished eating after 30 min, they were allowed to continue until they felt full (this did not occur during the study). Appetite questions were then asked at 30-min intervals for 5 h until immediately prior to consumption of the second ad libitum meal at 17.30 (*T* = 390). For this meal, an ad libitum portion of pasta with tomato and cheese sauce was offered followed by an ad libitum portion of lemon cake, following the same protocol as ad libitum meal one. Immediately after consumption of the meal, questionnaires on satiety were completed, after which participants were free to leave the Nutrition Unit. Upon completion of the study, participants received an honorarium to compensate them for their time.

To control for possible carryover effects, the order in which the participants received the three portions of almonds was counterbalanced. A Latin square design was used to ensure that all six possible sequences of presentation of the three portions of almonds occurred an equal number of times. However, perfect counterbalancing could only be achieved with *N* of 36, and 32 participants were recruited for the study, which resulted in four of the sequences of presentation occurring five times and two sequences (0, 42, 28 and 0, 28, 42 g) six times.

### Foods

For breakfast, participants were given foods that matched their habitual breakfast as closely as possible, usually toast or cereal plus milk. The same breakfast was given to each volunteer on the three test days. This created a self-regulated standardised baseline, meaning that all participants felt satiated to their usual level after breakfast. This avoided variation at the start and minimised inter-variation throughout the study period. Participants were given their habitual morning drink (tea/coffee/water, 200 ml) with breakfast to avoid caffeine withdrawal effects.

The almond (Almond Board of California) mid-morning snack was given to volunteers in weighed portions (0, 28 or 42 g), served raw and whole, and presented alongside 100 ml of water. On the control day (0 g almonds), participants were seated in the test product consumption area for the same period as when they received the test portions, in order to ensure that similar conditions were maintained throughout the study. The two different test quantities of almonds assessed were selected to be typical of normal consumption in a free-living situation. Due to the negative control (no almonds), the volunteers were not blinded to the test conditions. The 28 and 42 g portions of almonds delivered 173 and 259 kcals, respectively. These additional calories were considered in the overall energy intake across the study day.

*Ad libitum* meal 1 consisted of ham and cheese sandwiches [prepared on-site using Sainsbury’s (UK) white part-baked baguettes, Flora spreadable butter, Sainsbury’s sliced ham and Sainsbury’s cheddar cheese], and strawberry yogurt (Yeo Valley, UK). *Ad libitum* meal 2 consisted of pasta with tomato and cheese sauce (prepared on-site using Dolmio tomato sauce, Sainsbury’s penne pasta, Sainsbury’s vegetable oil and Sainsbury’s mozzarella cheese), and lemon cake slices (Mr Kipling, UK). The ad libitum meals were served in excess, more than can be reasonably consumed by an adult volunteer in a single sitting, and in all cases the meals were not finished by the volunteers. The sandwiches were cut into uneven shapes and served in foil trays and the yogurt in large bowls. The pasta meal was served in foil trays, and the cake was cut into small pieces. The foods were presented in this way to avoid suggesting portion sizes to the participants. Participants were asked to eat until they felt comfortably full and to then stop eating when they reached this stage. One hundred millilitre water was provided with each meal, and participants were instructed to consume all of the water. The energy content of the two ad libitum meals was calculated from the calorie content of the ingredients, and total energy intake was calculated by weighing the meals before and after consumption and converting the weight consumed into kcal. Participants were asked to record their liking of the breakfast, test foods and the two ad libitum meals after consumption, in order to identify any potentially confounding effects of meal palatability. Food liking was measured using VAS ratings in the format of “How much do you like the <food> overall?”; end-anchored with “not at all” and “very much”.

### Appetite ratings

Subjective ratings of appetite were recorded from 08.15 until 18.00 at regular intervals during the test days, as described in the protocol section. Responses were recorded with electronic VAS on hand-held computers (iPAQs), which prompted participants for a response at regular intervals in a pre-programmed manner. The scales were anchored at the low end with the lowest intensity feelings (e.g. extremely low) and with opposing terms at the high end (e.g. extremely high). Participants indicated on a 64-mm scale line the place that best reflected their feelings at that moment, and this was transformed into a score between 0 and 100. Scores were collected so that volunteers could not refer to their previous ratings of satiety. The questions asked as part of the Leatherhead test battery included: How hungry are you? How full are you? How satiated are you? How strong is your desire to eat? How much do you think you could eat right now? Would you like to eat something sweet? Would you like to eat something salty? Would you like to eat something savoury? Would you like to eat something fatty? Hunger and fullness were considered the main appetite ratings of interest.

### Statistical analysis

The study power calculation was based on within-subject differences in VAS appetite ratings reported by Flint et al. [21]. To detect a mean difference of 10 % using a repeated measures design with a power of 80 %, 32 participants were calculated as sufficient.

Statistical analysis was performed using STATA, version 12 (StataCorp LP, College Station, Texas, US), IBM SPSS version 21 and GraphPad Prism, version 5 (GraphPad Software Inc., La Jolla, USA) for Windows. Normality of the data was studied using gamma-3 and gamma-4 distribution parameters. Uncorrected *p* values are reported and results are reported as mean ± SEM. A *p* value lower than 0.05 was considered to be significant.

The main outcome measures, energy intake at each ad libitum meal and total energy intake on each test day, were compared over the three test days using repeated measures ANOVAs, with test day (0, 28 and 42 g almond snack) as the within-subject factor. The main subjective appetite ratings of interest were hunger and fullness. To assess how the almond snacks impacted on appetite sensations generally over the test days, total area under the curve (AUC) values for hunger and fullness were calculated using the trapezoidal rule [22] and compared across test days. To explore whether the almond mid-morning snack resulted in different patterns of reported hunger and fullness, repeated measures ANOVAs assessed hunger/fullness ratings from baseline to pre-ad libitum meal 1 (Interval 1 *T* = 0, 15, 30, 45, 60, 75, 90) and then from post-ad libitum meal 1 to pre-ad libitum meal 2 (Interval 2 *T* = 120, 150, 180, 210, 240, 270, 300, 330, 360, 390), with test day (0, 28 and 42 g almond snack) and time as the within-subject factors in all models. Differences at baseline for interval 1, defined as the time point prior to almond consumption (*T* = 0), and for interval 2, defined as the point immediately after lunch (*T* = 120), were analysed. Hunger [*T* = 0: *F*(2, 62) = 1.36; *p* = 0.27; *T* = 120: *F*(2, 62) = 0.65; *p* = 0.49] and fullness [*T* = 0: *F*(2, 62) = 1.36; *p* = 0.27; *T* = 120: *F*(2, 62) = 0.11; *p* = 0.90] ratings did not differ at these baselines. Because there were no significant baseline differences and because it is not possible to covary baseline ratings in within-subject ANOVA analyses, raw hunger and fullness data were analysed. For all analyses, since a dose-dependent effect of almonds was predicted, within-subject linear contrasts are reported for test day, with paired *t* tests used to identify significant differences between the three test days.

## Results

### Ad libitum intake

Table 2 shows participants’ energy intake at breakfast, the mid-morning almond snack and at each of the ad libitum meals. Participants’ energy intake at ad libitum meal 1 decreased in a dose-dependent manner in response to the almond snack [*F*(1, 31) = 47.3; *p* < 0.0001]: compared with the no almond test day, significantly less lunch was consumed on the 28 g (*p* = 0.016) and 42 g (*p* < 0.001) almond test days, and significantly less lunch was consumed after the 42 g portion compared to the 28 g portion (*p* = 0.005). The relationship between almond snack and intake at ad libitum meal 2 was also linear [*F*(1, 31) = 16.5; *p* < 0.001]: compared with the no almond test day, significantly less dinner was consumed on the 42 g test day (*p* < 0.001) but not 28 g test day (*p* = 0.28), and significantly less dinner was consumed after the 42 g portion compared with the 28 g portion (*p* = 0.047). Despite participants consuming an extra 173 and 259 kcals mid-morning on the 28 and 42 g almond test days, respectively, there were no significant differences in total energy intake (breakfast + snack + ad libitum meal 1 + ad libitum meal 2) across the three test days [*F*(1, 31) = 0.8; *p* = 0.38], indicating that participants appropriately compensated in a dose-dependent manner for the calories they consumed as almonds at the mid-morning snack.

**Table 2:** Energy intake (kcal) at the ad libitum meals and total energy intake

Almond test portion (g)	Breakfast	Snack	Ad libitum meal 1	Ad libitum meal 2	Total energy intake
0	344.5 ± 21.2	0	764.1 ± 23.3	1,060.0 ± 41.7	2,168.5 ± 59.7
28	343.9 ± 20.5	173	698.0 ± 31.3^a,b^	1,002.1 ± 36.0^b^	2,216.8 ± 63.6
42	341.9 ± 20.4	259	622.06 ± 30.2^a,b^	907.1 ± 36.3^a,b^	2,130.3 ± 51.5

Values are expressed as mean ± SEM

^a^Significantly different from control (0 g)

^b^Significantly different from the other test day

### Appetite ratings

Analysis of AUC data for *T* = 0–*T* = 390 (6.5 h) (Table 3) indicated that participants’ fullness levels throughout the test days showed a linear relationship with the amount of almonds they had consumed as a mid-morning snack [*F*(1, 30) = 9.2; *p* = 0.005]. They reported being more full on the 42 g day than on the 28 g day (*p* = 0.03) and on the no almond day (*p* = 0.005). Fullness levels did not differ overall on the 28 g day compared with the no almond day (*p* = 0.16). Overall AUC hunger levels also depended on almond intake [*F*(1, 30) = 11.2; *p* = 0.002]: on the 42 g day, participants reported being less hungry than on the no almond day (*p* = 0.002), but equally hungry as the 28 g day (*p* = 0.18). On the 28 g day, participants reported being significantly less hungry than on the no almond day (*p* = 0.025). To explore whether these significant differences in rated appetite were apparent throughout the test days or whether they depended on time of day, data were analysed with time as a factor.

**Table 3:** AUC (390 min) values ± SEM for fullness and hunger across the three test days

Almond test portion (g)	Fullness	Hunger
0	34,711 ± 1,203	23,854 ± 1,354
28	36,677 ± 1,589^b^	20,614 ± 1,499^a^
42	39,085 ± 1,773^a,b^	19,207 ± 1,497^a^

^a^Significantly different from control (0 g)

^b^Significantly different from the other test day

**Fig. 1:**
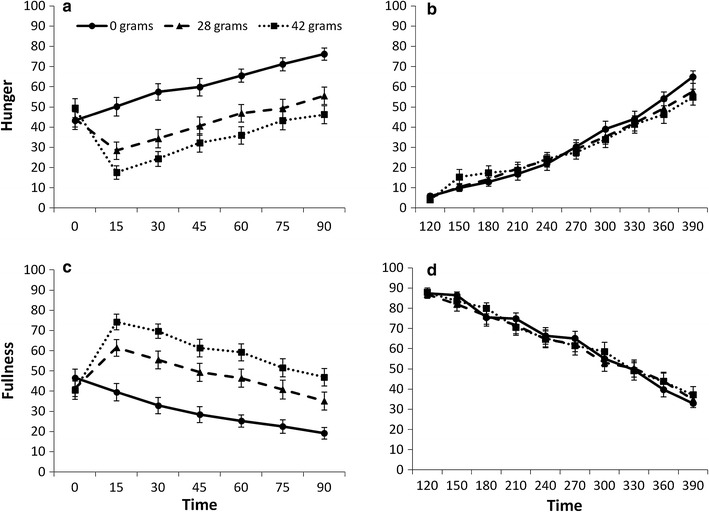
VAS scores of hunger (**a,** **b**) and fullness (**c**, **d**) rated from pre mid-morning snack to pre-ad libitum meal 1 (minutes 0–90: **a** and **c**) and from post-ad libitum meal 1 to pre-ad libitum meal 2 (minutes 120–390: **b** and **d**). Ratings were made on days when 28 g (*triangles*) and 42 g (*squares*) of almonds were consumed as a mid-morning snack, and on a 0 g almond control day (*circles*). From minutes 0–90, the mid-morning almond snack significantly influenced hunger and fullness ratings (both *p* < 0.0001)

Figure 1a and c shows appetite ratings during Interval 1 (pre-snack to pre-ad libitum meal 1: *T* = 0–90), which were dependent on the almond portion [Hunger *F*(1, 31) = 43.52; *p* < 0.0001; Fullness *F*(1, 31) = 44.05; *p* < 0.0001]. On the no almond day, during this interval, participants were more hungry than on the 28 g (*p* < 0.0001) and 42 g days (*p* < 0.0001) and more hungry on the 28 g day than on the 42 g day (*p* = 0.023). These differences appeared immediately after consumption of the mid-morning snack (*T* = 15) and were maintained in the period up to consumption of ad libitum meal 1 [*F*(12, 372) = 10.54; *p* < 0.0001]. Fullness ratings mirrored these results: participants were less full on the 0 g day than on the 28 g (*p* < 0.0001) and 42 g (*p* < 0.0001) days and less full on the 28 g day than on the 42 g day (*p* = 0.004). The increased fullness on the 28 and 42 g days appeared immediately after consumption of the mid-morning snack (*T* = 15), and this was maintained in the period up to consumption of ad libitum meal 1 [*F*(12, 372) = 11.30; *p* < 0.0001].

Figure 1b and d shows appetite ratings during Interval 2 (post-ad libitum meal 1 to pre-ad libitum meal 2 *T* = 120–390), which were similar on all three test days [Hunger *F*(1, 30) = 0.46; *p* = 0.50; Fullness F(1, 30) = 0.04; *p* = 0.85]. Moreover, patterns of these ratings during this interval were also similar over the three test days [Hunger *F*(18, 540) = 1.82; *p* = 0.10; Fullness *F*(18, 540) = 0.72; *p* = 0.69].

### Food evaluations

Rated liking did not significantly differ across the test days for breakfast [*F*(2, 62) = 2.62; *p* = 0.08], ad libitum meal 1 [*F*(2, 62) = 0.28; *p* = 0.76] and ad libitum meal 2 [*F*(2, 62) = 0.48; *p* = 0.64]. Liking for the 28 and 42 g portions of almonds did not significantly differ [*t*(31) = 0.30; *p* = 0.77].

## Discussion

This study assessed the short-term satiating effects of a mid-morning almond snack in healthy females. Results indicated a portion-dependent effect of almonds on appetite over the test days, with participants’ subjective reports of appetite and subsequent ad libitum food intake being dependent on the amount of almonds they had consumed mid-morning. Participants compensated well for the calories consumed as almonds, indicating that snacking on this food is not likely to increase total energy consumption over a day.

Previous studies of healthy people’s acute response to almond intake have examined glycaemic changes [16, 17] or self-report measures [18]; this is the first study to objectively examine how behaviour is affected. The volunteers adjusted their food intake in response to the almond snack at both lunch (ad libitum meal 1) and, to a lesser extent, at dinner (ad libitum meal 2). These effects are, for the most part, consistent with the impact the almond snack had on appetite sensations during the test days. Overall, the participants’ sensations of hunger and fullness across the test days depended on the amount of almonds consumed as a mid-morning snack. However, these effects were time dependent. Appetite ratings in the period between the almond snack and lunch depended on the amount of almonds consumed, with appetite suppressed to a greater extent the larger the portion of almonds; this might have been mediated by changes in gastrointestinal peptide release, though this was not assessed in this study. Whereas subjective appetite ratings in the interval between lunch and dinner did not depend on the portion of almonds consumed mid-morning, despite intake at dinner being lower when 42 g of almonds were consumed compared to 28 and 0 g. That lower intake at dinner on the 42 g day is not easily explained by increased levels of satiety in the run up to this meal points to other factors affecting eating behaviour. These might include the perception and memory of consuming the larger almond portion [23] or the perceived palatability of the test meals on this day, though this is not supported by the liking data. Alternatively, the VAS method of assessing changes in satiety might not have been sufficiently sensitive to pick up subtle differences in satiety during this interval. Future studies of healthy people assessing short-term biomarkers of satiety alongside behavioural measures would shed light on the mechanisms by which almond intake affects eating behaviour beyond the initial postprandial phase.

The present findings are consistent with longer-term studies of almond consumption indicating that regular almond intake is not a risk factor for weight gain [11–14]. Thus, the strong effect of almonds on the short-term experience of satiety might explain why frequent consumption of this energy-dense food has little impact on body weight. These longer-term studies also suggest that there is no adaptation effect when almonds are consumed regularly, so it might be predicted that the observed effects on short-term satiety are unlikely to diminish following habitual snacking on almonds. A related alternate explanation for the negligible effect of regular almond intake on body weight is that this food increases resting energy expenditure [12]. Whether such metabolic changes contributed to the almond-dependent reductions in intake found in this study is unknown.

In the present study, a control snack food of equal energy and volume to the almond snacks was not tested. This limits interpretation of the finding that an almond snack increased satiety in a dose-dependent manner since the effect could relate to ingestion of energy and not almonds per se. Another limiting factor is that habitual almond intake of the volunteers was not controlled for, which could have influenced satiety responses to this food on the test days. Furthermore, although the results of this study are representative of the population in which the research was carried out (i.e. women aged over 35 with a healthy body weight), this was a just convenience sample and results may not necessarily be replicated in other populations. For example, it is not known whether the ability to accurately compensate for an almond snack is compromised in overweight or obese people. Another consideration is that in order to empirically assess satiety responses, the timing of meals and the test environment was carefully controlled; thus, care must be taken when applying the findings to a free-living situations.

## Conclusions

This study indicates that adding almonds to the diet as a mid-morning snack is likely to increase satiety responses in a portion-dependent manner, leading to appropriate reductions in subsequent food intake so that total energy intake over the day is not increased. Almonds are energy and micronutrient dense, and they are also the tree nut highest in protein and fibre, which may account for their high satiety value. Since almonds are both nutritionally rich and satiating, they could be considered a snack food choice appropriate for a healthy diet.
